# Tau Propagation as a Diagnostic and Therapeutic Target for Dementia: Potentials and Unanswered Questions

**DOI:** 10.3389/fnins.2019.01274

**Published:** 2019-12-13

**Authors:** Shuko Takeda

**Affiliations:** Department of Clinical Gene Therapy, Graduate School of Medicine, Osaka University, Suita, Japan

**Keywords:** tau, propagation, dementia, Alzheimer’s disease, treatment, diagnosis

## Abstract

A unique clinical course of Alzheimer’s disease (AD), beginning with memory deficit as the earliest symptom, is well-correlated with a progressive pattern of intracellular aggregates of tau (neurofibrillary tangles), which spread from the medial temporal lobe to other brain areas in a stereotypical manner. Recent findings from basic research using *in vitro* and *in vivo* models demonstrated that pathological forms of extracellular tau can be taken up by cells and induce intracellular tau aggregates. On the basis of these neuropathological observations and experimental findings, the “tau propagation hypothesis” has been proposed, in which the stereotypical spreading of the tau pathology observed in the brain of AD patients can be explained by the interneuron transfer of the pathological form of tau. The concept of tau propagation remains controversial, and many unsolved questions exist; however, it has been attracting attention as a potential therapeutic target for halting AD progression. This article reviews the recent findings regarding the tau propagation hypothesis, including the basic concept and evidence of interneuron tau transfer, potentials as a diagnostic and therapeutic target, and unsolved questions for a better understanding of tau propagation.

## Introduction

Tau, a microtubule-associated protein mainly expressed in neurons, is involved in polymerizing microtubules and maintaining microtubule stability under physiological conditions (Grundke-Iqbal et al., 1986; Lewis and Dickson, 2016; Wang and Mandelkow, 2016). The majority of soluble tau is bound to microtubules, stabilizing them by shifting the equilibrium from free tubulin and microtubules toward polymerization. The physiological functions of tau are highly regulated by a wide range of posttranslational modifications, including phosphorylation, acetylation, glycation, isomerization, nitration, SUMOylation, and ubiquitination (Morris et al., 2011). The alteration of these modifications can affect tau functions and potentially lead to pathological conditions. Hyperphosphorylation of tau causes its detachment from microtubules, thereby impairing the axonal stability and trafficking necessary for normal neuronal activities (Morris et al., 2011).

Research indicates that the interaction of tau with microtubules is not as stable as previously believed (Janning et al., 2014), and tau exerts diverse functions that interact with multiple binding partners (Morris et al., 2011). Using single-molecule tracking experiments, Janning et al. (2014) showed that tau interacts with microtubules in a highly dynamic manner, with dwell time in the milliseconds range. The dynamic behavior of tau may underlie the regulation of microtubule dynamics and other functions coordinated with multiple binding partners. Tau interacts with plasma membrane-binding protein annexins, which may contribute to the enrichment and distribution of tau intracellularly and contribute to tau’s release from the cells (Gauthier-Kemper et al., 2018). The rapid, dynamic behavior of tau may be essential for its non-microtubule-related functions and its interactions with multiple binding partners in the cell (Morris et al., 2011).

**FIGURE 1:**
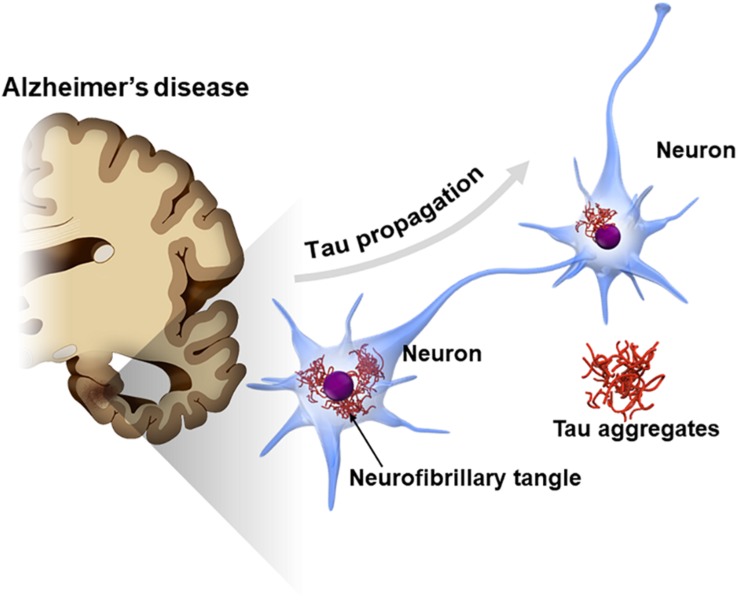
Propagation of tau pathology in Alzheimer’s disease (AD) brain. The tau pathology in the AD brain [neurofibrillary tangles (NFTs)] is known to spread along a neural network in a stereotypical manner. Interneuron transfer of the pathological form of tau may underlie the stereotypical progression of AD neuropathology (tau propagation hypothesis).

Tau is also known as a major component of neurofibrillary tangles (NFTs), one of the cardinal pathological features in Alzheimer’s disease (AD) (Hyman, 1997; Serrano-Pozo et al., 2011). A cross-sectional neuropathological study showed that the tau pathology of AD typically spreads from one area to another in a stereotypical pattern along a neural network (Braak and Braak, 1991). In early stage AD, NFTs appear in the transentorhinal cortex in the medial temporal lobe and later spread across the entire cerebral cortex via the hippocampal areas (Braak staging of NFT) (Braak and Braak, 1991). The mechanism underlying the stereotypical progression pattern of NFT in AD has not been elucidated; however, findings from recent studies suggest that tau pathology potentially spreads by the interneuronal transfer of the pathological from of tau (Figure 1; Hyman, 2014; Mudher et al., 2017; Gibbons et al., 2019). This phenomenon, called “tau propagation,” gains attention as a pathological hypothesis explaining the reason why AD progresses over time and also as a new therapeutic target for AD.

## Tau Pathology in the Brain of Patients With Dementia

### Tauopathy and Isoforms of Tau

A class of neurodegenerative disorders characterized by intracellular aggregates of tau in the brain, including AD, Pick’s disease, corticobasal degeneration, progressive supranuclear palsy, and argyrophilic grain disease, are collectively called tauopathy. Six isoforms of tau are known to be present in the human adult brain, and, in terms of the number of microtubule-binding repeats, tau isoforms are divided into two groups: 3-repeat and 4-repeat tau. A distinct pattern of tau accumulation is observed in each tauopathy: tau aggregates are present in either or both forms of tau isoforms – for example, 3-repeat tau in Pick’s disease; 4-repeat tau in corticobasal degeneration, progressive supranuclear palsy, and argyrophilic grain disease; and both 3-repeat and 4-repeat tau in AD (Lewis and Dickson, 2016). The differences in biochemical characteristics, including isoforms, of accumulated tau, are closely related to the pattern of progression of the tau pathology in each tauopathy (initial region and/or patterns of subsequent progression), which is important in understanding the tau propagation hypothesis.

### Progression Pattern of Tau Pathology in the Brain of AD Patients

Neurofibrillary tangles in AD patients appear first in the transentorhinal cortex or the entorhinal cortex in the medial temporal lobe (Braak stages I and II), then gradually progress to the hippocampal region (Braak stages III and IV), and finally involve the association neocortex or the primary areas of the neocortex (Braak stages V and VI) (Serrano-Pozo et al., 2011). This pattern of NFT progression closely resembles the clinical course of AD, which starts with severe memory deficit and slowly progresses to another cognitive dysfunction, indicating that the spread of the tau pathology is deeply associated with neurological dysfunction (Qian et al., 2017; DeVos et al., 2018). Since the progression of the tau pathology appears to spread along neuroanatomical connections, in other words, a brain region to another via axonal projections, the tau propagation hypothesis has been proposed, in which the pathological form of tau transfers between neurons (Clavaguera et al., 2013; Walker et al., 2013).

## Tau Propagation

### Tau Propagation *in vitro*

*In vitro* experiments performed by Frost et al. (2009) showed that a tau seed added to a culture medium can be taken up into cells via endocytosis and form new intracellular aggregates of tau. This finding provided theoretical evidence for the interneuronal transfer of tau as a mechanism underlying tau propagation.

Following the paper by Frost et al. (2009) multiple research groups reported on the mechanisms of the cellular uptake of extracellular tau and subsequent intracellular aggregation. A better understanding of the biochemical features of the tau involved in propagation is important in developing therapeutic strategies (Sanders et al., 2014; Panza et al., 2016). The first report by Frost et al. (2009) showed that the fibrillar form of tau is more easily taken up into the cells than the monomeric form of tau. On the other hand, Mirbaha et al. (2015) reported that the tau trimer is the minimal unit that is capable of inducing intracellular aggregates of tau. Wu et al. (2013) and Takeda et al. (2015) performed experiments using a unique chamber for neuronal cell culture with a microfluidic chip demonstrating that the tau oligomer is more easily propagated than the tau monomer. Another report demonstrated that the monomeric form of tau can mediate tau propagation (Michel et al., 2014). To date, which form of tau is really involved in interneuronal propagation remains controversial (Table 1).

**TABLE 1:** The nature of tau species involved in propagation.

**The nature of tau species**	**Origin of tau seeds**	**Experimental model**	**References**
Tau monomer	Recombinant tau	Tau uptake in cell culture model	Michel et al. (2014)
Tau trimers	Recombinant tauBrain-derived tau from AD brain	Tau uptake in cell culture model	Mirbaha et al. (2015)
Tau oligomers (dimer/trimer)	Brain-derived tau from AD brain	Intracereberal injections of tau seeds in mouse model	Lasagna-Reeves et al. (2012)
Low-molecular-weight aggregates (spherical oligomers with diameters ranging from 10 to 30 nm)	Recombinant tau	Trans-synaptic transfer of tau in microfluidic devices	Wu et al. (2013)
Phosphorylated large tau oligomers (>10 mers)	Brain-derived tau from tau-transgenic mice	Tau uptake in cell culture modelIntracereberal injections of tau seeds in mouse model	Jackson et al. (2016)
Phosphorylated high-molecular-weight tau (>600 kDa)	Brain-derived tau from AD brainBrain-derived tau from tau-transgenic mice	Tau uptake in cell culture modelTrans-synaptic transfer of tau in microfluidic devicesIntracereberal injections of tau seeds in mouse model	Takeda et al. (2015)
Tau aggregates	Recombinant tau	Tau uptake in cell culture model	Frost et al. (2009)
Tau aggregates	Exosomal tau from primary neurons or cerebrospinal fluid of AD patients	Tau uptake in cell culture modelTrans-synaptic transfer of tau in microfluidic devices	Wang et al. (2017)
Tau fibrils	Recombinant tau	Intracereberal injections of tau seeds in mouse model	Iba et al. (2013)
Tau fibrils	Brain-derived tau from AD brain	Intracereberal injections of tau seeds in mouse model	Guo et al. (2016)

*AD, Alzheimer’s disease.*

The aggregation and propagation properties of tau also depend on its isoforms (Nonaka et al., 2010; Dinkel et al., 2011). Variations of the tau isoform can affect posttranslational modification, such as the fragmentation and phosphorylation of tau, which can potentially modify intracellular uptake and aggregation capability (Despres et al., 2017). The propagation activity of tau can also be affected by the type of genetic mutation or the origin of tau (synthetic or brain-derived tau protein) (Lewis and Dickson, 2016).

### Tau Propagation in Animal Models

Clavaguera et al. (2009) reported that intracellular tau aggregates can be induced in the mouse brain that was injected with seed-competent tau aggregates, demonstrating that tau propagation may occur even *in vivo*. In this experiment, brain homogenates from tau-transgenic mice that overexpress the mutant form of tau in the brain were injected into another mouse brain that does not have the tau pathology; 12 months later, tau aggregates were observed in the neurons of the recipient mouse brain.

In 2012, three research groups independently reported almost similar mouse models of tau propagation (de Calignon et al., 2012; Harris et al., 2012; Liu et al., 2012). Transgenic mice overexpressing human mutant tau (P301L) only in the entorhinal cortex were generated using a region-specific promoter, and the neuropathological changes were examined over time up to 2 years. First, the tau pathology appeared in the entorhinal cortex (where the human mutant tau was overexpressed) at the age of 12 months and then appeared in the neurons of the dentate gyrus, which has strong neuroanatomical connection to the entorhinal cortex but is not supposed to express the human mutant tau, at the age of about 18 months. These experiments demonstrated that, although there are some problems/limitations including the region specificity of the promoter, tau pathologies that comprise the human mutant tau in neurons of the entorhinal cortex can potentially spread likely via synaptic transmission to neurons in the dentate gyrus, a major target of axonal projections.

Along the same line, Iba et al. (2013) and Ahmed et al. (2014) conducted *in vivo* experiments wherein the fibrillar form of recombinant tau, or tau-transgenic mouse brain-derived tau, was injected into a specific region of the mouse brain that does harbor the tau pathology. Newly induced tau pathology appeared in the brain regions along a neural network from the injection site. The region where the tau pathology appeared was not related to the distance from the tau seed injection site but to a neuroanatomical connection (along the axonal projection), implying that tau propagation possibly occurs via synapses.

### Mechanism of Tau Propagation

**FIGURE 2:**
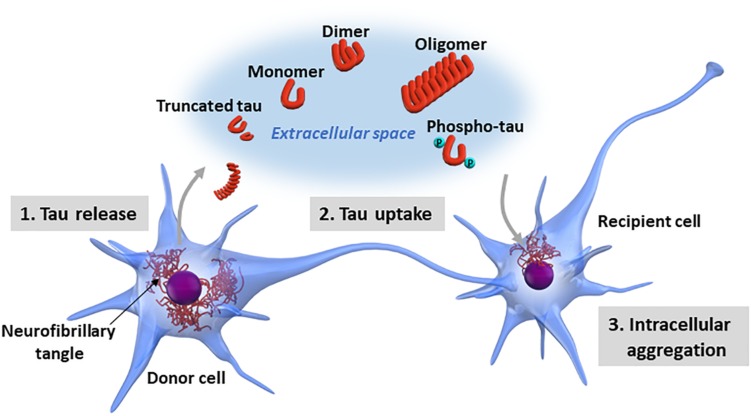
Neuron-to-neuron transfer of tau. The processes involved in tau propagation can be divided into three basic steps: (1) the pathological form of tau is released into the extracellular space from the donor cell; (2) the pathological tau released into the extracellular space is taken up by recipient cells; and (3) the pathological tau taken up into the recipient cells forms new intracellular aggregates. Tau exists in various forms in terms of biochemical property, including monomeric, oligomeric, truncated, and phosphorylated forms. Additionally, tau may undergo a wide range of posttranslational modifications, including acetylation, glycation, isomerization, nitration, SUMOylation, and ubiquitination or a mixture of these modifications. It is still largely unknown which forms of tau are released into the extracellular space and involved in tau propagation.

Although researchers have demonstrated that certain forms of pathological tau have the property of transmission between neurons (Calafate et al., 2015), the molecular mechanisms underlying tau propagation are still largely unknown. The interneuronal propagation of tau is divided into three steps: the intracellular pathological tau (1) is released into the extracellular space, (2) is taken up by recipient cells, and (3) forms new intracellular aggregates in the recipient cells (Figure 2).

Regarding tau release into the extracellular space, passive leakage from degenerated cells and tau dissociation from ghost tangles likely contribute to it. Recent studies have shown a possibility that physiological active tau release could occur without neurodegeneration (Yamada et al., 2011, 2014; Pooler et al., 2013), which may be involved in tau propagation (Wu et al., 2016). The cellular uptake of extracellular tau can potentially be mediated by endocytosis (Holmes et al., 2013) occurring on the cell surface or during synaptic transmission. A recent study by Falcon et al. (2018) showed that seed-competent tau enters cells via clathrin-independent endocytosis and escape from damaged endomembranes into the cytosol, triggering cytosolic tau aggregation. The galectin-8-dependent autophagy system mediated the delivery of tau seeds from the endo-lysosomal pathway to the cytosol, implying a role for autophagy in intracellular tau aggregation and propagation.

Tau propagation is known to occur in both directions (retrograde and anterograde) along a neural network (Ahmed et al., 2014; Takeda et al., 2015), which indicates that tau propagation does not necessarily occur only via synaptic transmission. Asai et al. and other researchers reported that exosomes are also involved in the mechanism that mediates interneuronal tau transfer (Asai et al., 2015; Wang et al., 2017).

### Propagation of Other Pathological Proteins

Researchers have reported on other proteins implicated in neurodegenerative diseases, including amyloid-β (Petkova et al., 2005; Watts et al., 2014; Sengupta et al., 2016; Condello and Stoehr, 2018; Ruiz-Riquelme et al., 2018) or α-synuclein (Dehay et al., 2016; McCann et al., 2016; Steiner et al., 2018), which show the key biochemical properties and capabilities of cell-to-cell propagation. Although they show distinct distributions in the human brain, misfolded forms of these pathological proteins may propagate through mechanisms similar to tau (Goedert et al., 2017). In the brains of AD patients, amyloid-β plaques develop in the basal temporal and orbitofrontal cortex in the early phase before reaching other areas, such as the neocortex, hippocampal formation, and basal ganglia, and then finally spreading to the lower brain stem. Inclusions of α-synuclein first develop in the peripheral nervous system and olfactory bulb, ascend to the brainstem and midbrain, and then spread to the basal forebrain and neocortex (Braak et al., 2003; Goedert et al., 2017). Distinct conformations of each pathological protein may determine the distribution pattern and speed of spread and may underlie the phenotypic diversity of neurodegenerative diseases. Although the molecular mechanisms mediating the propagation of pathological proteins have not been fully understood, they can be specific targets for therapeutic interventions.

## Unsolved Questions Regarding Tau Propagation Hypothesis

Tau propagation is an intriguing pathological hypothesis for explaining the fundamental characteristics of dementia that progresses over time, but many questions regarding its mechanisms or the validity of the hypothesis still remain unsolved.

It is necessary to carefully examine how exactly the tau pathology in cultured cells and brains of animal models reflects the tau pathology observed in the human brain. So far, there is no clear evidence showing biochemical and biological similarities between the structures of tau aggregates in cultured cells or mouse brains and those of the NFTs in AD patients.

In humans, the accumulation of tau aggregates is accompanied by various cognitive dysfunctions associated with neuronal death; however, with some exceptions, the tau pathology induced in cell lines or mouse brains is often not accompanied by neuronal death or dysfunction. This may be because the tau seeds used in experimental models for propagation study are not identical to the species that exert neurotoxicity in human tauopathies. Moreover, scientists do not know which species of tau is really involved in neurotoxicity (Ballatore et al., 2007). Propagation property and neurotoxicity may be independent features of distinct forms of tau. Additionally, most experiments based on cell or animal models with overexpression of the mutant form of tau may not accurately reflect the brain pathology in patients with sporadic AD without genetic mutation.

There are some issues regarding the validity of the concept of tau propagation. In the tau propagation hypothesis, the medial temporal lobe is considered an initial site of origin for tau propagation starting in the brain of AD patients. However, the tau pathology in AD is known to begin in areas such as the dorsal raphe nuclei (Grinberg et al., 2009) and the locus coeruleus (Satoh and Iijima, 2019) or it can also begin in the area of the neocortex rather than the medial temporal lobe (Braak and Braak, 1991; Lewis and Dickson, 2016). The dorsal raphe nuclei and locus coeruleus are connected with the transentorhinal cortex where tau pathology develops in the early stages of AD. These nuclei may be affected by tau pathology even before the transentorhinal cortex, and they may act as an initiation site for the subsequent spread of tau pathology throughout the brain (Grinberg et al., 2009; Satoh and Iijima, 2019).

The heterogenicity of the distribution of the AD tau pathology should also be taken into consideration when understanding the propagation hypothesis; the initial site of origin for tau propagation may not be limited to only one site, but multiple brain regions could be the starting points of propagation instead. Tau aggregates also appear in glial cells in some types of tauopathies, but the applicability of the tau propagation hypothesis to glial tau pathology remains unclear.

Some fundamental issues have not been solved, including why conformational changes and aggregation initiate at the initial site of origin for tau propagation. What is the most upstream trigger of tau aggregation in the initial site? In human tauopathy, even in cases caused by genetic mutation in which all cells are supposed to uniformly possess the same mutation, a striking laterality of neuropathology is occasionally observed. Can the tau propagation hypothesis explain the asymmetricity observed in some cases of tauopathy? These unsolved questions need to be discussed and answered for a better understanding of tau propagation.

## Future Directions: Toward Diagnosis and Treatment Based on the Tau Propagation Hypothesis

A stereotypical pattern of the progression of the tau pathology in the brains of patients with sporadic AD may be explained by the tau propagation hypothesis, which can potentially lead to the development of diagnostic and treatment strategies for AD (Sigurdsson, 2016; Takeda, 2019).

Takeda et al. (2016) examined the biological and biochemical properties of brain extracellular tau from various sources including lumbar cerebrospinal fluid (CSF) from AD patients. They found that the bioactive tau species involved in propagation is present in the CSF from AD patients, and its concentrations were significantly higher than those in control subjects or patients with frontotemporal dementia. This finding suggests that the tau species involved in propagation could be useful as a biomarker for AD, specifically for monitoring tau propagation activity.

The tau propagation hypothesis, based on the concept that the clinical progression of AD is linked with the spreading of the tau pathology, supports the idea that clearing the tau involved in propagation may slow the spread of the tau pathology and possibly of cognitive decline (Nicholls et al., 2017; Nobuhara et al., 2017). The potential efficacy of antibody-based therapeutics targeting tau has been demonstrated in several studies using animal models (Yanamandra et al., 2013; Pedersen and Sigurdsson, 2015), although it is unclear how an antibody can exert its therapeutic efficacy against an intracellular protein such as tau. In this regard, the tau propagation hypothesis may provide a rationale for the antibody-based strategy targeting tau propagation that could be mediated by extracellular tau, which can be captured and neutralized by an anti-tau antibody; the antibody does not need to be incorporated into the cell to exert its therapeutic efficacy (Takeda, 2019).

Identifying the most efficient epitope should be critical in therapeutic development for tau-based immunotherapy. So far, various antibodies against distinct epitopes of tau have been tested (Takeda, 2019). Some antibodies successfully ameliorated the cognitive deficit and neuropathology in mouse models. Nobuhara et al. (2017) used a cell culture model to assess the effect of multiple anti-tau antibodies on tau propagation to identify suitable target epitopes for blocking tau uptake and propagation. The blocking efficacy varied depending on the epitope that each anti-tau antibody targeted, and the antibody against the N-terminal and phospho-site of tau showed the most effectiveness (Nobuhara et al., 2017). Other processes involved in tau propagation, including tau release, cellular uptake, and intracellular aggregation, could be therapeutic targets for halting the spread of the tau pathology, which need further investigation.

## Conclusion

This article reviewed the recent findings regarding the tau propagation hypothesis, namely, the basic concept and evidence of tau propagation, potential as a diagnostic and therapeutic target, and unsolved questions. The hypothesis has been attracting considerable attention in the research field of ADbecause it may explain the stereotypical progression of the tau pathology in the brain of AD patients and provides a rationale for tau-based therapies. Numerous questions regarding the detailed molecular mechanisms underlying the tau propagation remain unanswered. Identifying the specific tau species involved in propagation, molecules mediating tau release and uptake, and surrogate markers for propagation activity should be the key research targets for tackling tau propagation.

## Author Contributions

The author confirms being the sole contributor of this work and has approved it for publication.

## Conflict of Interest

The author declares that the research was conducted in the absence of any commercial or financial relationships that could be construed as a potential conflict of interest.

## References

[B1] Ahmed Z. Cooper J. Murray T. K. Garn K. McNaughton E. Clarke H. (2014). A novel in vivo model of tau propagation with rapid and progressive neurofibrillary tangle pathology: the pattern of spread is determined by connectivity, not proximity. *Acta Neuropathol.* 127 667–683. 10.1007/s00401-014-1254-6 24531916 PMC4252866

[B2] Asai H. Ikezu S. Tsunoda S. Medalla M. Luebke J. Haydar T. (2015). Depletion of microglia and inhibition of exosome synthesis halt tau propagation. *Nat. Neurosci.* 18 1584–1593. 10.1038/nn.4132 26436904 PMC4694577

[B3] Ballatore C. Lee V. M. Trojanowski J. Q. (2007). Tau-mediated neurodegeneration in Alzheimer’s disease and related disorders. *Nat. Rev. Neurosci.* 8 663–672. 10.1038/nrn2194 17684513

[B4] Braak H. Braak E. (1991). Neuropathological stageing of Alzheimer-related changes. *Acta Neuropathol.* 82 239–259. 10.1007/bf00308809 1759558

[B5] Braak H. Rub U. Gai W. P. Del Tredici K. (2003). Idiopathic Parkinson’s disease: possible routes by which vulnerable neuronal types may be subject to neuroinvasion by an unknown pathogen. *J. Neural Transm.* 110 517–536. 10.1007/s00702-002-0808-2 12721813

[B6] Calafate S. Buist A. Miskiewicz K. Vijayan V. Daneels G. de Strooper B. (2015). Synaptic contacts enhance cell-to-cell Tau pathology propagation. *Cell Rep.* 11 1176–1183. 10.1016/j.celrep.2015.04.043 25981034

[B7] Clavaguera F. Bolmont T. Crowther R. A. Abramowski D. Frank S. Probst A. (2009). Transmission and spreading of tauopathy in transgenic mouse brain. *Nat. Cell Biol.* 11 909–913. 10.1038/ncb1901 19503072 PMC2726961

[B8] Clavaguera F. Lavenir I. Falcon B. Frank S. Goedert M. Tolnay M. (2013). “Prion-like” templated misfolding in tauopathies. *Brain Pathol.* 23 342–349. 10.1111/bpa.12044 23587140 PMC8028860

[B9] Condello C. Stoehr J. (2018). Abeta propagation and strains: implications for the phenotypic diversity in Alzheimer’s disease. *Neurobiol. Dis.* 109 191–200. 10.1016/j.nbd.2017.03.014 28359847

[B10] de Calignon A. Polydoro M. Suarez-Calvet M. William C. Adamowicz D. H. Kopeikina K. J. (2012). Propagation of tau pathology in a model of early Alzheimer’s disease. *Neuron* 73 685–697. 10.1016/j.neuron.2011.11.033 22365544 PMC3292759

[B11] Dehay B. Vila M. Bezard E. Brundin P. Kordower J. H. (2016). Alpha-synuclein propagation: new insights from animal models. *Mov. Disord.* 31 161–168. 10.1002/mds.26370 26347034

[B12] Despres C. Byrne C. Qi H. Cantrelle F. X. Huvent I. Chambraud B. (2017). Identification of the Tau phosphorylation pattern that drives its aggregation. *Proc. Natl. Acad. Sci. U.S.A.* 114 9080–9085. 10.1073/pnas.1708448114 28784767 PMC5576827

[B13] DeVos S. L. Corjuc B. T. Oakley D. H. Nobuhara C. K. Bannon R. N. Chase A. (2018). Synaptic Tau seeding precedes Tau pathology in human Alzheimer’s disease brain. *Front. Neurosci.* 12:267. 10.3389/fnins.2018.00267 29740275 PMC5928393

[B14] Dinkel P. D. Siddiqua A. Huynh H. Shah M. Margittai M. (2011). Variations in filament conformation dictate seeding barrier between three- and four-repeat tau. *Biochemistry* 50 4330–4336. 10.1021/bi2004685 21510682

[B15] Falcon B. Noad J. McMahon H. Randow F. Goedert M. (2018). Galectin-8-mediated selective autophagy protects against seeded tau aggregation. *J. Biol. Chem.* 293 2438–2451. 10.1074/jbc.M117.809293 29282296 PMC5818177

[B16] Frost B. Jacks R. L. Diamond M. I. (2009). Propagation of tau misfolding from the outside to the inside of a cell. *J. Biol. Chem.* 284 12845–12852. 10.1074/jbc.M808759200 19282288 PMC2676015

[B17] Gauthier-Kemper A. Suarez Alonso M. Sundermann F. Niewidok B. Fernandez M. P. Bakota L. (2018). Annexins A2 and A6 interact with the extreme N terminus of tau and thereby contribute to tau’s axonal localization. *J. Biol. Chem.* 293 8065–8076. 10.1074/jbc.RA117.000490 29636414 PMC5971446

[B18] Gibbons G. S. Lee V. M. Y. Trojanowski J. Q. (2019). Mechanisms of cell-to-cell transmission of pathological Tau: a review. *JAMA Neurol.* 76 101–108. 10.1001/jamaneurol.2018.2505 30193298 PMC6382549

[B19] Goedert M. Masuda-Suzukake M. Falcon B. (2017). Like prions: the propagation of aggregated tau and alpha-synuclein in neurodegeneration. *Brain* 140 266–278. 10.1093/brain/aww230 27658420

[B20] Grinberg L. T. Rub U. Ferretti R. E. Nitrini R. Farfel J. M. Polichiso L. (2009). The dorsal raphe nucleus shows phospho-tau neurofibrillary changes before the transentorhinal region in Alzheimer’s disease. A precocious onset? *Neuropathol. Appl. Neurobiol.* 35 406–416. 10.1111/j.1365-2990.2009.00997.x 19508444

[B21] Grundke-Iqbal I. Iqbal K. Quinlan M. Tung Y. C. Zaidi M. S. Wisniewski H. M. (1986). Microtubule-associated protein tau. A component of Alzheimer paired helical filaments. *J. Biol. Chem.* 261 6084–6089. 3084478

[B22] Guo J. L. Narasimhan S. Changolkar L. He Z. Stieber A. Zhang B. (2016). Unique pathological tau conformers from Alzheimer’s brains transmit tau pathology in nontransgenic mice. *J. Exp. Med.* 213 2635–2654. 10.1084/jem.20160833 27810929 PMC5110027

[B23] Harris J. A. Koyama A. Maeda S. Ho K. Devidze N. Dubal D. B. (2012). Human P301L-mutant tau expression in mouse entorhinal-hippocampal network causes tau aggregation and presynaptic pathology but no cognitive deficits. *PLoS One* 7:e45881. 10.1371/journal.pone.0045881 23029293 PMC3454317

[B24] Holmes B. B. DeVos S. L. Kfoury N. Li M. Jacks R. Yanamandra K. (2013). Heparan sulfate proteoglycans mediate internalization and propagation of specific proteopathic seeds. *Proc. Natl. Acad. Sci. U.S.A.* 110 E3138–E3147. 10.1073/pnas.1301440110 23898162 PMC3746848

[B25] Hyman B. T. (1997). The neuropathological diagnosis of Alzheimer’s disease: clinical-pathological studies. *Neurobiol. Aging* 18 S27–S32. 9330982 10.1016/s0197-4580(97)00066-3

[B26] Hyman B. T. (2014). Tau propagation, different tau phenotypes, and prion-like properties of tau. *Neuron* 82 1189–1190. 10.1016/j.neuron.2014.06.004 24945760

[B27] Iba M. Guo J. L. McBride J. D. Zhang B. Trojanowski J. Q. Lee V. M. (2013). Synthetic tau fibrils mediate transmission of neurofibrillary tangles in a transgenic mouse model of Alzheimer’s-like tauopathy. *J. Neurosci.* 33 1024–1037. 10.1523/JNEUROSCI.2642-12.2013 23325240 PMC3575082

[B28] Jackson S. J. Kerridge C. Cooper J. Cavallini A. Falcon B. Cella C. V. (2016). Short fibrils constitute the major species of seed-competent Tau in the brains of mice transgenic for human P301S Tau. *J. Neurosci.* 36 762–772. 10.1523/jneurosci.3542-15.2016 26791207 PMC4719013

[B29] Janning D. Igaev M. Sundermann F. Bruhmann J. Beutel O. Heinisch J. J. (2014). Single-molecule tracking of tau reveals fast kiss-and-hop interaction with microtubules in living neurons. *Mol. Biol. Cell* 25 3541–3551. 10.1091/mbc.E14-06-1099 25165145 PMC4230615

[B30] Lasagna-Reeves C. A. Castillo-Carranza D. L. Sengupta U. Guerrero-Munoz M. J. Kiritoshi T. Neugebauer V. (2012). Alzheimer brain-derived tau oligomers propagate pathology from endogenous tau. *Sci. Rep.* 2:700. 10.1038/srep00700 23050084 PMC3463004

[B31] Lewis J. Dickson D. W. (2016). Propagation of tau pathology: hypotheses, discoveries, and yet unresolved questions from experimental and human brain studies. *Acta Neuropathol.* 131 27–48. 10.1007/s00401-015-1507-z 26576562

[B32] Liu L. Drouet V. Wu J. W. Witter M. P. Small S. A. Clelland C. (2012). Trans-synaptic spread of tau pathology in vivo. *PLoS One* 7:e31302. 10.1371/journal.pone.0031302 22312444 PMC3270029

[B33] McCann H. Cartwright H. Halliday G. M. (2016). Neuropathology of alpha-synuclein propagation and braak hypothesis. *Mov. Disord.* 31 152–160. 10.1002/mds.26421 26340605

[B34] Michel C. H. Kumar S. Pinotsi D. Tunnacliffe A. St George-Hyslop P. Mandelkow E. (2014). Extracellular monomeric tau protein is sufficient to initiate the spread of tau protein pathology. *J. Biol. Chem.* 289 956–967. 10.1074/jbc.M113.515445 24235150 PMC3887218

[B35] Mirbaha H. Holmes B. B. Sanders D. W. Bieschke J. Diamond M. I. (2015). Tau Trimers are the minimal propagation unit spontaneously internalized to seed intracellular aggregation. *J. Biol. Chem.* 290 14893–14903. 10.1074/jbc.M115.652693 25887395 PMC4463437

[B36] Morris M. Maeda S. Vossel K. Mucke L. (2011). The many faces of tau. *Neuron* 70 410–426. 10.1016/j.neuron.2011.04.009 21555069 PMC3319390

[B37] Mudher A. Colin M. Dujardin S. Medina M. Dewachter I. Alavi Naini S. M. (2017). What is the evidence that tau pathology spreads through prion-like propagation? *Acta Neuropathol. Commun.* 5:99. 10.1186/s40478-017-0488-7 29258615 PMC5735872

[B38] Nicholls S. B. DeVos S. L. Commins C. Nobuhara C. Bennett R. E. Corjuc D. L. (2017). Characterization of TauC3 antibody and demonstration of its potential to block tau propagation. *PLoS One* 12:e0177914. 10.1371/journal.pone.0177914 28531180 PMC5439699

[B39] Nobuhara C. K. DeVos S. L. Commins C. Wegmann S. Moore B. D. Roe A. D. (2017). Tau antibody targeting pathological species blocks neuronal uptake and interneuron propagation of Tau *in vitro*. *Am. J. Pathol.* 187 1399–1412. 10.1016/j.ajpath.2017.01.022 28408124 PMC5455060

[B40] Nonaka T. Watanabe S. T. Iwatsubo T. Hasegawa M. (2010). Seeded aggregation and toxicity of {alpha}-synuclein and tau: cellular models of neurodegenerative diseases. *J. Biol. Chem.* 285 34885–34898. 10.1074/jbc.M110.148460 20805224 PMC2966103

[B41] Panza F. Solfrizzi V. Seripa D. Imbimbo B. P. Lozupone M. Santamato A. (2016). Tau-Centric targets and drugs in clinical development for the treatment of Alzheimer’s disease. *Biomed. Res. Int.* 2016:3245935. 10.1155/2016/3245935 27429978 PMC4939203

[B42] Pedersen J. T. Sigurdsson E. M. (2015). Tau immunotherapy for Alzheimer’s disease. *Trends Mol. Med.* 21 394–402. 25846560 10.1016/j.molmed.2015.03.003

[B43] Petkova A. T. Leapman R. D. Guo Z. Yau W. M. Mattson M. P. Tycko R. (2005). Self-propagating, molecular-level polymorphism in Alzheimer’s beta-amyloid fibrils. *Science* 307 262–265. 10.1126/science.1105850 15653506

[B44] Pooler A. M. Phillips E. C. Lau D. H. Noble W. Hanger D. P. (2013). Physiological release of endogenous tau is stimulated by neuronal activity. *EMBO Rep.* 14 389–394. 10.1038/embor.2013.15 23412472 PMC3615658

[B45] Qian J. Hyman B. T. Betensky R. A. (2017). Neurofibrillary tangle stage and the rate of progression of Alzheimer symptoms: modeling using an autopsy cohort and application to clinical trial design. *JAMA Neurol.* 74 540–548. 10.1001/jamaneurol.2016.5953 28288263 PMC5547572

[B46] Ruiz-Riquelme A. Lau H. H. C. Stuart E. Goczi A. N. Wang Z. Schmitt-Ulms G. (2018). Prion-like propagation of beta-amyloid aggregates in the absence of APP overexpression. *Acta Neuropathol. Commun.* 6:26. 10.1186/s40478-018-0529-x 29615128 PMC5883524

[B47] Sanders D. W. Kaufman S. K. DeVos S. L. Sharma A. M. Mirbaha H. Li A. (2014). Distinct tau prion strains propagate in cells and mice and define different tauopathies. *Neuron* 82 1271–1288. 10.1016/j.neuron.2014.04.047 24857020 PMC4171396

[B48] Satoh A. Iijima K. M. (2019). Roles of tau pathology in the locus coeruleus (LC) in age-associated pathophysiology and Alzheimer’s disease pathogenesis: potential strategies to protect the LC against aging. *Brain Res.* 1702 17–28. 10.1016/j.brainres.2017.12.027 29274876

[B49] Sengupta U. Nilson A. N. Kayed R. (2016). The role of amyloid-beta oligomers in toxicity, propagation, and immunotherapy. *EBioMedicine* 6 42–49. 10.1016/j.ebiom.2016.03.035 27211547 PMC4856795

[B50] Serrano-Pozo A. Frosch M. P. Masliah E. Hyman B. T. (2011). Neuropathological alterations in Alzheimer disease. *Cold Spring Harb. Perspect. Med.* 1:a006189. 10.1101/cshperspect.a006189 22229116 PMC3234452

[B51] Sigurdsson E. M. (2016). Tau immunotherapy. *Neurodegener. Dis.* 16 34–38. 10.1159/000440842 26551002 PMC4777344

[B52] Steiner J. A. Quansah E. Brundin P. (2018). The concept of alpha-synuclein as a prion-like protein: ten years after. *Cell Tissue Res.* 373 161–173. 10.1007/s00441-018-2814-1 29480459 PMC6541204

[B53] Takeda S. (2019). Progression of Alzheimer’s disease, tau propagation, and its modifiable risk factors. *Neurosci. Res.* 141 36–42. 10.1016/j.neures.2018.08.005 30120962

[B54] Takeda S. Commins C. DeVos S. L. Nobuhara C. K. Wegmann S. Roe A. D. (2016). Seed-competent high-molecular-weight tau species accumulates in the cerebrospinal fluid of Alzheimer’s disease mouse model and human patients. *Ann. Neurol.* 80 355–367. 10.1002/ana.24716 27351289 PMC5016222

[B55] Takeda S. Wegmann S. Cho H. DeVos S. L. Commins C. Roe A. D. (2015). Neuronal uptake and propagation of a rare phosphorylated high-molecular-weight tau derived from Alzheimer’s disease brain. *Nat. Commun.* 6:8490. 10.1038/ncomms9490 26458742 PMC4608380

[B56] Walker L. C. Diamond M. I. Duff K. E. Hyman B. T. (2013). Mechanisms of protein seeding in neurodegenerative diseases. *JAMA Neurol.* 70 304–310. 23599928 10.1001/jamaneurol.2013.1453 PMC3665718

[B57] Wang Y. Balaji V. Kaniyappan S. Kruger L. Irsen S. Tepper K. (2017). The release and trans-synaptic transmission of Tau via exosomes. *Mol. Neurodegener.* 12:5. 10.1186/s13024-016-0143-y 28086931 PMC5237256

[B58] Wang Y. Mandelkow E. (2016). Tau in physiology and pathology. *Nat. Rev. Neurosci.* 17 5–21. 26631930 10.1038/nrn.2015.1

[B59] Watts J. C. Condello C. Stohr J. Oehler A. Lee J. DeArmond S. J. (2014). Serial propagation of distinct strains of Abeta prions from Alzheimer’s disease patients. *Proc. Natl. Acad. Sci. U.S.A.* 111 10323–10328. 10.1073/pnas.1408900111 24982139 PMC4104857

[B60] Wu J. W. Herman M. Liu L. Simoes S. Acker C. M. Figueroa H. (2013). Small misfolded Tau species are internalized via bulk endocytosis and anterogradely and retrogradely transported in neurons. *J. Biol. Chem.* 288 1856–1870. 10.1074/jbc.M112.394528 23188818 PMC3548495

[B61] Wu J. W. Hussaini S. A. Bastille I. M. Rodriguez G. A. Mrejeru A. Rilett K. (2016). Neuronal activity enhances tau propagation and tau pathology in vivo. *Nat. Neurosci.* 19 1085–1092. 10.1038/nn.4328 27322420 PMC4961585

[B62] Yamada K. Cirrito J. R. Stewart F. R. Jiang H. Finn M. B. Holmes B. B. (2011). In vivo microdialysis reveals age-dependent decrease of brain interstitial fluid tau levels in P301S human tau transgenic mice. *J. Neurosci.* 31 13110–13117. 10.1523/JNEUROSCI.2569-11.2011 21917794 PMC4299126

[B63] Yamada K. Holth J. K. Liao F. Stewart F. R. Mahan T. E. Jiang H. (2014). Neuronal activity regulates extracellular tau in vivo. *J. Exp. Med.* 211 387–393. 10.1084/jem.20131685 24534188 PMC3949564

[B64] Yanamandra K. Kfoury N. Jiang H. Mahan T. E. Ma S. Maloney S. E. (2013). Anti-tau antibodies that block tau aggregate seeding in vitro markedly decrease pathology and improve cognition in vivo. *Neuron* 80 402–414. 10.1016/j.neuron.2013.07.046 24075978 PMC3924573

